# Sleep Duration and the Risk of Mortality From Stroke in Japan: The Takayama Cohort Study

**DOI:** 10.2188/jea.JE20140272

**Published:** 2016-03-05

**Authors:** Toshiaki Kawachi, Keiko Wada, Kozue Nakamura, Michiko Tsuji, Takashi Tamura, Kie Konishi, Chisato Nagata

**Affiliations:** 1 Department of Epidemiology and Preventive Medicine, Gifu University Graduate School of Medicine, Gifu, Japan; 1 岐阜大学大学院医学研究科疫学予防医学分野; 2 Department of Food and Nutrition, Gifu City Women’s College, Gifu, Japan; 2 岐阜市立女子短期大学食物栄養学科; 3 Department of Food and Nutrition, Nagoya Women’s University, Nagoya, Japan; 3 名古屋女子大学食物栄養学科

**Keywords:** sleep duration, cardiovascular, stroke, ischemic stroke, cohort studies, 睡眠時間, 心血管, 脳卒中, 脳梗塞, コホート研究

## Abstract

**Background:**

Few studies have assessed the associations between sleep duration and stroke subtypes. We examined whether sleep duration is associated with mortality from total stroke, ischemic stroke, and hemorrhagic stroke in a population-based cohort of Japanese men and women.

**Methods:**

Subjects included 12 875 men and 15 021 women aged 35 years or older in 1992, who were followed until 2008. The outcome variable was stroke death (ischemic stroke, hemorrhagic stroke, and total stroke).

**Results:**

During follow-up, 611 stroke deaths (354 from ischemic stroke, 217 from hemorrhagic stroke, and 40 from undetermined stroke) were identified. Compared with 7 h of sleep, ≥9 h of sleep was significantly associated with an increased risk of total stroke and ischemic stroke mortality after controlling for covariates. Hazard ratios (HRs) and 95% confidence intervals (CIs) were 1.51 (95% CI, 1.16–1.97) and 1.65 (95% CI, 1.16–2.35) for total stroke mortality and ischemic stroke mortality, respectively. Short sleep duration (≤6 h of sleep) was associated with a decreased risk of mortality from total stroke (HR 0.77; 95% CI, 0.59–1.01), although this association was of borderline significance (*P* = 0.06). The trends for total stroke and ischemic stroke mortality were also significant (*P* < 0.0001 and *P* = 0.0002, respectively). There was a significant risk reduction of hemorrhagic stroke mortality for ≤6 h of sleep as compared with 7 h of sleep (HR 0.64; 95% CI, 0.42–0.98; *P* for trend = 0.08). The risk reduction was pronounced for men (HR 0.31; 95% CI, 0.16–0.64).

**Conclusions:**

Data suggest that longer sleep duration is associated with increased mortality from total and ischemic stroke. Short sleep duration may be associated with a decreased risk of mortality from hemorrhagic stroke in men.

## Abstract (ja)

**【背景】:**

睡眠時間とタイプ別脳卒中の関係についての研究は少ない。我々は、日本人一般集団からなるコホートにおいて睡眠時間と全脳卒中およびタイプ別脳卒中の死亡リスクとの関連について検討した。

**【方法】:**

対象は、1992年に35歳以上で、2008年まで追跡された男性12,875名、女性15,021名である。エンドポイントは脳卒中死亡（脳梗塞、脳出血、全脳卒中）とした。

**【結果】:**

追跡中に611人の脳卒中死亡（脳梗塞354名、脳出血217名、その他40名）を認めた。共変量を調整後、7時間睡眠と比較し、9時間以上の睡眠は全脳卒中および脳梗塞の死亡リスクが有意に増加した。そのハザード比と95％信頼区間はそれぞれ1.51 (95%信頼区間1.16-1.97) および 1.65 (95%信頼区間1.16-2.35)であった。短時間睡眠(6時間以下)では、全脳卒中の死亡リスクの減少(ハザード比0.77, 95%信頼区間0.59-1.01)を示したが、有意差は境界域(P=0.06)であった。全脳卒中と脳梗塞の死亡リスクの量反応関係は、有意であった (それぞれP<0.0001 および P=0.0002)。脳出血において、7時間睡眠に比較し、6時間以下の睡眠では死亡リスクが有意に減少(ハザード比0.64 (95%信頼区間0.42-0.98)し、特に男性において、顕著なリスク低下（ハザード比0.31 (95%CI 0.16-0.64)）を認めた。

**【結論】:**

全脳卒中および脳梗塞において、長時間睡眠は死亡リスク増加との関係を示した。脳出血において、男性の短時間睡眠は死亡リスク低下との関係が示唆された。

## INTRODUCTION

Stroke is one of the main causes of death in Western countries and in the Asia-Pacific region, including Japan. In particular, ischemic stroke accounts for more than 70% of all stroke cases in Western countries and about 74% of cases in Japan.^1^ Compared with the incidence of myocardial infarction, the incidence of stroke is high in Japan,^2^ and stroke is a disease that causes many to become bedridden. Because it generates a high burden and societal cost, effective primary preventive strategies are needed.^3^ Besides the established risk factors, such as age, hypertension,^4^^,^^5^ diabetes,^6^^,^^7^ dyslipidemia,^8^ smoking,^9^ and atrial fibrillation,^10^ environmental and lifestyle factors might play an important role in the etiology of strokes. Several previous studies have suggested an association between sleep duration and cardiovascular disease (CVD), including stroke.^11^^–^^22^ A meta-analysis by Cappuccio et al^23^ reported that both short and long sleep durations were associated with increased incidence or mortality of stroke; hazard ratios (HRs) were 1.15 (95% confidence interval [CI], 1.00–1.37) for short sleep durations and 1.65 (95% CI, 1.45–1.87) for long sleep durations. However, the number of component studies was small (n = 5). Furthermore, studies on the subtypes of stroke are also scarce.^20^^,^^24^^,^^25^

Therefore, we investigated whether sleep duration is associated with total stroke and subtypes of stroke in a cohort of Japanese men and women (the Takayama cohort study).^26^

## METHODS

### Study population

Subjects in this study were cohort members from a population-based cohort study conducted in Takayama City, Gifu, Japan. The methodology of the study design has been described previously.^26^ Eligible participants were all non-hospitalized residents of Takayama who were aged 35 years or older. In 1992, 14 427 men and 17 125 women completed a self-administered baseline questionnaire that included questions on demographic characteristics, smoking and drinking habits, diet, exercise, and medical and reproductive histories. The participation rate was 85.3% after excluding incomplete or unreliable responses to the dietary questionnaire. For this analysis, we also excluded subjects who had reported on the baseline questionnaire that they had a past history of cancer (186 men and 540 women) or coronary heart disease and stroke (886 men and 861 women). Furthermore, 480 men and 703 women who did not answer the question regarding sleep duration were also excluded. The remaining 27 896 subjects (12 875 men and 15 021 women) were included in the present analysis. This study was approved by the ethics board of the Gifu University Graduate School of Medicine.

### Measurement of baseline variables

Descriptive parameters are shown as means (standard deviations) or proportions (%) at baseline. Although height and weight were self-reported, we previously tested the validity of the data in a sample from this community. High correlations of measured height and weight with reported ones were noted (*r* = 0.93 and 0.97, respectively). Smoking status was also based on self-report. Physical activity was assessed from the average hours per week spent performing various kinds of activities during the previous year. The time spent per week performing an activity of specific intensity was multiplied by its corresponding energy expenditure requirements, expressed as a metabolic equivalent, and summed to obtain a score (metabolic equivalents-h/week). Details, including the validity of this method, have been described elsewhere.^27^ Alcohol consumption was assessed using a food frequency questionnaire. The questions on alcohol use include six types: *sake*, beer, light beer, *shochu* (a type of distilled liquor commonly consumed in Japan), wine, and hard liquor. The amount of ethanol was calculated by multiplying the frequency of alcohol use by the ethanol content of the specified portion size.

### Follow-up and outcome

All deaths in Takayama and their causes during the follow-up period (1992–2008) were identified from death certificates provided by the Legal Affairs Bureau, Japan. The causes of death were coded according to the International Classification of Diseases, 9th and 10th Revisions (ICD-9 and -10). Information on subjects who had moved away from Takayama during the study period was obtained from the residential registers of the city. The primary endpoint for our analysis was stroke mortality (ICD-9 codes 434–48 and ICD-10 codes I60–I69). Strokes were classified as ischemic strokes (ICD-9 codes 434 and ICD-10 codes I63 and I69.3), intracerebral hemorrhages (ICD-9 codes 431 and ICD-10 codes I61 and I69.1), subarachnoid hemorrhages (ICD-9 code 430 and ICD-10 codes I60 and I69.0), and strokes of undetermined types.

### Statistical analysis

For each subject, the number of person-years of follow-up was calculated from the study entry (September 1992) to the date of death, the date on which the person moved away from Takayama City, or the end of the study (March 2008), whichever came first. During the study period, 941 (6.5%) men and 971 (5.7%) women moved away from Takayama City. Of these, the moving date was unknown for 104 (0.7%) men and 147 (0.9%) women. They were censored at the latest date when they were known to reside in the city.

The associations between subjects’ characteristics and sleep duration were assessed using the analysis of variance or the Cochran-Mantel-Haenszel chi-square test after controlling for age. We used Cox proportional hazards regression analysis to estimate the HRs and the 95% CIs for death from stroke for the sleep duration categories (≤6, 7, 8, and ≥9 h/day). The reference category was 7 h of sleep duration. For stroke, the analyses were done for total stroke, ischemic stroke, and hemorrhagic (subarachnoid hemorrhage and cerebral hemorrhage) stroke, as the numbers of cases in each subtype were small. The selection of potential confounders was based primarily on a priori consideration of their association with short and long sleep durations and stroke as well as the change in risk estimates before and after adjustment. Variables adjusted for in the multivariate model included age, level of education, marital status, body mass index (BMI), smoking status, alcohol consumption, and history of hypertension and diabetes. The significance of interaction by sex was tested by including cross-product terms of sleep duration (as a continuous variable) and sex (as a dichotomous variable).

In sensitivity analysis, we repeated the analysis after excluding subjects who were deceased or censored during the first 5 years of the follow-up and those who had histories of hypertension or diabetes at baseline. In addition, those who had history of tuberculosis, blood transfusion, or gastrectomy were also excluded. All statistical analyses were performed using SAS statistical software (version 9.2.0; SAS Institute, Inc., Cary, NC, USA).

## RESULTS

Baseline characteristics of the study population according to sleep duration categories are given in Table 1. Age ranged from 35 to 97 years. Approximately 40% of subjects, both men and women, reported sleeping 7 h per night. Subjects reporting longer sleep duration had lower alcohol consumption and were more likely to be lean, smokers, not married, less educated, and less physically active. They were also more likely to have reported histories of hypertension, diabetes, tuberculosis, blood transfusion, and gastrectomy.

**Table 1.** Baseline characteristics of the study population according to sleep duration

	Sleep duration (h/day)				*P*-value^a^

	≤6	7	8	≥9	
All					
Number of subjects	6693	11 087	7594	2522	
Age, years^b^	53.0 (11.6)	52.1 (11.3)	55.4 (12.6)	63.3 (15.0)	<0.0001
Body mass index, kg/m^2 b^	22.3 (2.9)	22.3 (2.8)	22.2 (2.8)	21.8 (3.2)	<0.0001
History of hypertension^c^	17.5	15.7	18.9	25.4	0.008
History of diabetes^c^	3.8	3.5	4.4	6.5	0.008
History of tuberculosis^c^	3.5	3.3	4.0	4.9	0.006
History of blood transfusion^c^	4.5	3.9	4.4	5.2	0.007
History of gastrectomy^c^	1.5	1.4	1.9	2.4	0.002
Alcohol consumption, g/day^b^	21.4 (34.7)	22.8 (33.7)	25.6 (36.6)	21.1 (38.1)	0.02
Physical activity score, METs·h/week^b^	23.4 (36.9)	23.9 (35.9)	23.1 (36.1)	19.4 (35.0)	<0.0001
Smoking status^b^					
never	57.1	51.9	46.7	44.0	
past	13.1	14.5	18.2	21.3	
current	29.8	33.6	35.1	34.6	0.0007
Length of education^c^					
≤11 years	58.8	55.2	65.4	79.4	
11–14 years	32.6	35.5	26.9	16.9	
≥15 years	8.6	9.3	7.8	3.7	<0.0001
Married^c^	81.7	86	83.5	71.9	<0.0001
Men					
Number of subjects	2428	5067	4005	1375	
Age, years^b^	52.3 (11.1)	51.6 (10.9)	54.4 (12.0)	61.3 (14.1)	<0.0001
Body mass index, kg/m^2 b^	22.8 (2.9)	22.6 (2.7)	22.4 (2.7)	21.9 (2.9)	<0.0001
History of hypertension^c^	20.1	16.8	18.9	24.2	0.004
History of diabetes^c^	5.9	5.3	6.1	7.1	0.46
History of tuberculosis^c^	4.5	3.9	4.4	5.6	0.09
History of blood transfusion^c^	3.8	3.3	3.9	4.3	0.41
History of gastrectomy^c^	2.8	2	2.7	3.1	0.03
Alcohol consumption, g/day^b^	43.6 (44.3)	41.0 (40.0)	42.1 (41.8)	43.5 (41.3)	<0.0001
Physical activity score, METs·h/week^b^	27.7 (43.2)	27.6 (41.3)	28.2 (41.8)	25.5 (41.1)	<0.0001
Smoking status^c^					
never	18.1	16.4	16.7	16.1	
past	27.5	26.6	29.2	32.3	
current	54.3	57.0	54.2	51.6	0.02
Length of education^c^					
≤11 years	53.2	49.4	59.5	76.8	
11–14 years	33.3	36.5	29.4	18.1	
≥15 years	13.6	14.2	11.1	5.0	<0.0001
Married^b^	89.8	92.4	92.8	88.0	<0.0001
Women					
Number of subjects	4265	6020	3589	1147	
Age, years^b^	53.5 (11.8)	52.4 (11.6)	56.6 (13.3)	65.6 (15.6)	<0.0001
Body mass index, kg/m^2 b^	22.0 (2.9)	22.0 (2.8)	20.0 (2.9)	21.7 (3.4)	0.41
History of hypertension^c^	16.0	14.8	18.8	26.9	0.11
History of diabetes^c^	2.7	2.1	2.5	5.8	0.0004
History of tuberculosis^c^	2.9	2.7	3.5	4.0	0.09
History of blood transfusion^c^	5.0	4.3	4.9	6.3	0.01
History of gastrectomy^c^	0.8	0.8	1.0	1.6	0.02
Alcohol consumption, g/day^b^	8.8 (18.1)	7.4 (15.2)	7.2 (15.9)	7.5 (20.7)	0.02
Physical activity score, METs·h/week^b^	20.9 (32.5)	20.7 (30.3)	17.4 (27.4)	12.2 (23.7)	<0.0001
Smoking status^c^					
never	81.1	83.7	83.1	80.8	
past	4.2	3.7	4.8	6.8	
current	14.8	12.6	12.1	12.4	0.0002
Length of education^c^					
≤11 years	62.0	60.2	72.0	82.5	
11–14 years	32.3	34.7	24.0	15.4	
≥15 years	5.8	5.1	4.0	2.2	<0.0001
Married^c^	77.0	80.6	73.0	52.4	0.0002

METs, metabolic equivalents.

^a^Adjusted for age. Additionally adjusted for sex for men and women combined.

^b^Mean (standard deviations).

^c^Percentage.

During the follow-up, there were 611 stroke deaths, including 354 (172 men and 182 women) from ischemic stroke, 217 (109 men and 108 women) from hemorrhagic stroke (intracerebral hemorrhage and subarachnoid hemorrhage), and 40 (15 men and 25 women) from undetermined stroke. Table 2 shows age-adjusted and multivariable hazard ratios of total stroke and the subtypes of stroke according to the sleep duration category. As the number of cases with undetermined type of stroke was small, the results for this type are not shown. Compared with 7 h of sleep, ≥9 h of sleep was significantly associated with an increased risk of mortality from total stroke and ischemic stroke in the entire sample; the HRs were 1.51 (95% CI, 1.16–1.97) and 1.65 (95% CI, 1.16–2.35), respectively. Short sleep duration (≤6 h of sleep) was associated with a decreased risk of mortality from total stroke (HR 0.77; 95% CI, 0.59–1.01), although this association was of borderline significance (*P* = 0.06). The trends of increasing risk associated with the longer sleep duration were significant for total stroke and ischemic stroke mortality (*P* < 0.0001 and *P* = 0.0002, respectively). A significant reduction in the risk of mortality from hemorrhagic stroke (HR 0.64; 95% CI, 0.42–0.98) was observed for ≤6 h of sleep, but the trend was not significant.

**Table 2.** Hazard ratios and 95% confidence intervals for mortality from stroke according to sleep duration

	Sleep duration (h/day)				*P-trend*

	≤6	7	8	≥9	
All					
Person-years	96 844	161 100	106 629	30 713	
Total stroke					
*Number of cases*	*88*	*167*	*194*	*162*	
Age-adjusted HR (95% CI)	0.79 (0.60–1.04)	1.00	1.14 (0.92–1.42)	1.57 (1.21–2.04)	*<0.0001*
Multivariate HR^a^ (95% CI)	0.77 (0.59–1.01)	1.00	1.13 (0.91–1.40)	1.51 (1.16–1.97)	*<0.0001*
Ischemic stroke					
*Number of cases*	*51*	*77*	*119*	*107*	
Age-adjusted HR (95% CI)	0.94 (0.64–1.36)	1.00	1.40 (1.04–1.89)	1.74 (1.22–2.46)	*0.0002*
Multivariate HR^a^ (95% CI)	0.93 (0.64–1.34)	1.00	1.33 (0.99–1.80)	1.65 (1.16–2.35)	*0.0002*
Hemorrhagic stroke					
*Number of cases*	*34*	*84*	*66*	*33*	
Age-adjusted HR (95% CI)	0.68 (0.44–1.03)	1.00	0.90 (0.64–1.27)	1.01 (0.64–1.62)	*0.07*
Multivariate HR^a^ (95% CI)	0.64 (0.42–0.98)	1.00	0.90 (0.64–1.26)	0.96 (0.60–1.54)	*0.08*
Men					
Person-years	33 889	71 272	55 416	17 008	
Total stroke					
*Number of cases*	*29*	*98*	*93*	*76*	
Age-adjusted HR (95% CI)	0.55 (0.37–0.84)	1.00	0.89 (0.67–1.18)	1.29 (0.95–1.77)	*0.0002*
Multivariate HR^a^ (95% CI)	0.51 (0.34–0.77)	1.00	0.88 (0.66–1.17)	1.23 (0.90–1.69)	*0.0003*
Ischemic stroke					
*Number of cases*	*19*	*45*	*57*	*51*	
Age-adjusted HR (95% CI)	0.73 (0.43–1.25)	1.00	1.06 (0.72–1.58)	1.39 (0.92–2.11)	*0.009*
Multivariate HR^a^ (95% CI)	0.67 (0.39–1.16)	1.00	1.04 (0.70–1.54)	1.35 (0.88–2.05)	*0.008*
Hemorrhagic stroke					
*Number of cases*	*9*	*53*	*34*	*13*	
Age-adjusted HR (95% CI)	0.34 (0.17–0.69)	1.00	0.72 (0.47–1.11)	0.70 (0.37–1.30)	*0.34*
Multivariate HR^a^ (95% CI)	0.31 (0.16–0.64)	1.00	0.70 (0.46–1.09)	0.63 (0.34–1.18)	*0.35*
Women					
Person-years	62 955	89 826	51 213	13 704	
Total stroke					
*Number of cases*	*59*	*69*	*101*	*86*	
Age-adjusted HR (95% CI)	1.08 (0.76–1.52)	1.00	1.52 (1.11–2.06)	1.97 (1.41–2.75)	*0.0003*
Multivariate HR^a^ (95% CI)	1.06 (0.75–1.50)	1.00	1.50 (1.10–2.04)	1.93 (1.38–2.70)	*0.0003*
Ischemic stroke					
*Number of cases*	*32*	*32*	*62*	*56*	
Age-adjusted HR (95% CI)	1.24 (0.76–2.02)	1.00	1.83 (1.19–2.81)	2.12 (1.34–3.35)	*0.008*
Multivariate HR^a^ (95% CI)	1.22 (0.75–1.99)	1.00	1.75 (1.14–2.68)	2.07 (1.30–3.27)	*0.008*
Hemorrhagic stroke					
*Number of cases*	*25*	*31*	*32*	*20*	
Age-adjusted HR (95% CI)	1.06 (0.62–1.79)	1.00	1.30 (0.79–2.13)	1.72 (0.95–3.12)	*0.15*
Multivariate HR^a^ (95% CI)	1.04 (0.61–1.76)	1.00	1.29 (0.78–2.12)	1.65 (0.91–2.99)	*0.15*

CI, confidence interval; HR, hazard ratio.

^a^Estimated hazard ratio after adjustments for age, education years, marital status, histories of hypertension and diabetes, body mass index, physical activity score, smoking status, and alcohol consumption (g/day). Additionally adjusted for sex for men and women combined.

The increases in the risk of mortality from total stroke and ischemic stroke associated with ≥9 h of sleep were not significant in men, but the trends were significant in both men and women. The associations between sleep duration and mortality from total stroke, ischemic stroke, and hemorrhagic stroke did not differ significantly between men and women (*P* values for interaction were 0.45, 0.47, and 0.35, respectively).

The sensitivity analysis revealed that the risk increases in mortality from total stroke and ischemic stroke for ≥9 h of sleep were somewhat attenuated when study subjects were restricted to those with better health (ie, without histories of hypertension, diabetes, tuberculosis, blood transfusion, and gastrectomy) (Table 3). Similar tendencies were observed after excluding deaths and censored cases during the first 5 years of follow-up. However, the trend remained significant in these analyses.

**Table 3.** Sensitivity analyses of the associations between sleep duration and the risk of mortality from stroke

	Sleep duration (h/day)				*P-trend*

	≤6	7	8	≥9	
Total stroke					
Model 1^a^					
Cases/Subjects	54/5348	98/9054	119/5927	84/1785	
Multivariate HR^b^ (95% CI)	0.81 (0.57–1.15)	1.00	1.20 (0.90–1.60)	1.40 (0.98–1.99)	*0.0007*
Model 2^c^					
Cases/Subjects	43/4930	86/8414	105/5422	70/1608	
Multivariate HR^b^ (95% CI)	0.76 (0.51–1.12)	1.00	1.18 (0.87–1.60)	1.24 (0.84–1.82)	*0.005*
Model 3^d^					
Cases/Subjects	77/6526	135/10 806	150/7247	98/2173	
Multivariate HR^b^ (95% CI)	0.82 (0.61–1.10)	1.00	1.14 (0.89–1.46)	1.44 (1.06–1.95)	*0.003*
Ischmeic stroke					
Model 1^a^					
Cases/Subjects	30/5348	94/9054	66/5927	56/1785	
Multivariate HR^b^ (95% CI)	0.95 (0.58–1.56)	1.00	1.34 (0.90–2.01)	1.60 (0.99–2.57)	*0.01*
Model 2^c^					
Cases/Subjects	21/4930	38/8414	56/5422	46/1608	
Multivariate HR^b^ (95% CI)	0.82 (0.47–1.45)	1.00	1.25 (0.81–1.93)	1.30 (0.77–2.19)	*0.03*
Model 3^d^					
Cases/Subjects	47/6526	68/10 806	97/7247	66/2173	
Multivariate HR^b^ (95% CI)	0.92 (0.60–1.37)	1.00	1.32 (0.95–1.83)	1.51 (1.01–2.34)	*0.053*
Hemorrhagic stroke					
Model 1^a^					
Cases/Subjects	21/5348	52/9054	45/5927	14/1785	
Multivariate HR^b^ (95% CI)	0.65 (0.38–1.12)	1.00	1.04 (0.65–1.54)	0.68 (0.34–1.32)	*0.43*
Model 2^c^					
Cases/Subjects	18/4930	45/8414	42/5422	12/1608	
Multivariate HR^b^ (95% CI)	0.69 (0.39–1.22)	1.00	1.06 (0.68–1.67)	0.64 (0.31–1.32)	*0.51*
Model 3^d^					
Cases/Subjects	27/6526	63/10 806	53/7247	24/2173	
Multivariate HR^b^ (95% CI)	0.68 (0.42–1.10)	1.00	0.98 (0.67–1.44)	1.06 (0.62–1.81)	*0.09*

CI, confidence interval; HR, hazard ratio.

^a^Excluding subjects with hypertension and diabetes.

^b^Estimated hazard ratio after adjustments for age, sex, education years, marital status, histories of hypertension and diabetes, body mass index, physical activity score, smoking status, and alcohol consumption (g/day).

^c^Excluding subjects with hypertension, diabetes, tuberculosis, gastrectomy, and blood transfusion.

^d^Excluding deaths and censored cases within the first 5 years.

## DISCUSSION

In this population-based prospective cohort study, we observed that long sleep duration (≥9 hours per day) was significantly associated with an increased risk of mortality from total stroke and ischemic stroke in men and women. To our knowledge, five prospective studies thus far have assessed the association between sleep duration and total stroke. The First National Health and Nutrition Examination Survey Epidemiologic Follow-up Study in the United States among 7844 men and women reported a significantly increased risk of total stroke (relative risk [RR] 1.5; 95% CI, 1.1–2.0) with sleep duration of >8 h compared with 6–8 h of sleep.^11^ The Japan Collaborative Cohort Study (JACC study) among 98 634 subjects reported a significantly increased total stroke mortality associated with long sleep duration (≥10 h) compared with 7 h of sleep in men (HR 1.66; 95% CI, 1.31–2.08) and women (HR 1.69; 95% CI, 1.29–2.20).^20^ Short sleep duration (≤4 h) was associated with a 1.7-fold increased risk of total stroke mortality in men, but this association was not statistically significant. The Jichi Medical School Cohort Study in Japan among 11 367 subjects did not find any significant association between total stroke mortality and short or long sleep durations.^22^ The national Finnish Population Survey on Risk Factors on Chronic, Noncommunicable Diseases study among 25 025 Finnish men and women observed that long sleep duration (≥10 h) was significantly associated with an increased risk of fatal and non-fatal strokes in women (HR 1.40; *P* = 0.05) compared with 7–8 h.^28^ The Singapore Chinese Health Study among 63 257 subjects observed that long (≥9 h) and short (≤5 h) sleep duration compared with 7 h of sleep duration were significantly associated with increased risk of total stroke mortality; the HRs were 1.54 (95% CI, 1.28–1.85) and 1.25 (95% CI, 1.05–1.50), respectively.^25^

Studies reporting the relationship between the subtypes of stroke and sleep duration are few. The Women’s Health Initiative Observational Cohort Study reported a significantly increased ischemic stroke risk associated with long sleep duration (>8 h) compared with 7 h of sleep (HR 1.70; 95% CI, 1.32–2.21). The risk increase with short sleep duration (≤6 h) was not statistically significant (HR 1.14; 95% CI, 0.97–1.33).^24^ In the JACC Study^20^ and the Singapore Chinese Health Study,^25^ the associations of sleep duration with ischemic stroke mortality were similar to those with total stroke mortality. In the former, long sleep duration (≥10 h) was significantly associated with increased ischemic stroke mortality in men (HR 1.58; 95% CI, 1.19–2.12) and women (HR 2.37; 95% CI, 1.70–3.32). In the latter, long sleep duration (≥9 h) and short sleep duration (≤5 h) were significantly associated with increased ischemic stroke mortality; HRs were 1.68 (95% CI, 1.36–2.06) and 1.37 (95% CI, 1.12–1.68), respectively. Both studies did not observe a significant association between sleep duration and hemorrhagic stroke mortality.

In general, previous studies have reported an increased risk for total stroke or ischemic stroke with a long duration of sleep. The present study confirmed a positive association between a long duration of sleep and mortality from total stroke and ischemic stroke. The association between a short duration of sleep and the risk of stroke has been equivocal. Some, but not all, studies showed increased risk for short duration of sleep.^24^^,^^25^ However, our data rather suggest a decreased risk of mortality from total stroke associated with short duration of sleep. This reduction reflected the decrease of mortality from hemorrhagic stroke associated with ≤6 h of sleep in men. The tests for linear trend would not assess the shape of the risk pattern, but higher mortality risks for total stroke and ischemic stroke were associated with longer duration of sleep. Nonetheless, the number of subjects with short durations of sleep (eg, ≤5 and 6 h) was small, which limited our ability to separately analyze the effect of ≤5 and 6 h of sleep. There is some evidence that long sleep durations were associated with increased risks for diabetes^29^ and hypertension,^30^ lipid abnormalities,^31^ elevated inflammation markers,^32^ and atrial fibrillation.^33^ These conditions could induce arteriosclerosis and atherosclerosis, which can lead to stroke or stroke death. However, we should mention that some studies reported that short sleep duration was also associated with hypertension^23^ and diabetes.^29^

Our current study has several methodological advantages. As the study was prospective, we were able to reduce the likelihood of recall bias and minimize selection bias. In addition, the participants of our study were residents of a community in Japan. The small loss among this cohort during the follow-up period and high participation rate at baseline were also advantages of our study.

On the other hand, our study had several limitations. First, sleep durations were self-reported. The study was unable to distinguish whether the times reported were actual physiologic sleep times or total time in bed. However, self-reported sleep duration has been demonstrated to yield valid results consistent with quantitative sleep assessments with actigraphy.^34^ Second, we could not measure blood pressure, which is the most important risk factor of stroke. Third, we had no information on the quality of sleep, such as insomnia or sleep apnea. Sleep apnea has been suggested to be associated with long sleep duration as well as stroke and CVD mortality.^35^^–^^39^ However, considering that being overweight is a strong determinant for sleep apnea and our study population was generally lean, the potential confounding effect of sleep apnea may not be great. In addition, BMI was included as a covariate in the models. Fourth, the sample size was limited, which precluded analyses in stroke subtypes with small numbers of deaths. Fifth, sleep duration was assessed only at baseline. Finally, because of the use of mortality data instead of incidence data, we were unable to distinguish whether sleep duration is a risk factor or a prognostic factor for stroke. Although the results were not changed greatly after excluding those who died during the first 5 years of follow-up, underlying disease or preclinical signs may have caused long sleep durations. As the inclusion of subjects with some illness somewhat attenuated the association between sleep duration and stroke mortality, we cannot deny a possible confounding effect of general health status.

In conclusion, our results suggest that longer sleep durations are associated with increased mortality from total stroke and ischemic stroke. Furthermore, short sleep duration may be associated with a decreased risk of mortality from hemorrhagic stroke in men. The causal effect association remains unclear. Larger and longer studies that include repeated measurements of sleep duration are encouraged.

## ONLINE ONLY MATERIAL

Abstract in Japanese.
