# Supplementary material for: Sleep Duration and the Risk of Mortality From Stroke in Japan: The Takayama Cohort Study
Source: J Epidemiol. 2016 Mar 5;26(3):123–30. doi: 10.2188/jea.JE20140272 (PMC4773488; doi:10.2188/jea.JE20140272)
Supplement: Abstract in Japanese. [file je-26-123-s001.pdf]

日本人における睡眠時間と脳卒中死亡リスク－高山コホート研究－

川地俊明[1]、和田恵子[1]、中村こず枝[2]、辻美智子[3]、田村高志[1]、小西　希恵[1]、永田知里[1]
[1]岐阜大学大学院医学研究科疫学予防医学分野
[2]岐阜市立女子短期大学食物栄養学科
[3]名古屋女子大学食物栄養学科

【背景】睡眠時間とタイプ別脳卒中の関係についての研究は少ない。我々は、日本人一般集団からなるコホートにおいて睡眠時間と全脳卒中およびタイプ別脳卒中の死亡リスクとの関連について検討した。
【方法】対象は、1992 年に 35 歳以上で、2008 年まで追跡された男性 12,875 名、女性 15,021 名である。エンドポイントは脳卒中死亡（脳梗塞、脳出血、全脳卒中）とした。
【結果】追跡中に 611 人の脳卒中死亡（脳梗塞 354 名、脳出血 217 名、その他 40 名）を認めた。共変量を調整後、7 時間睡眠と比較し、9 時間以上の睡眠は全脳卒中および脳梗塞の死亡リスクが有意に増加した。そのハザード比と 95%信頼区間はそれぞれ 1.51（95%信頼区間 1.16-1.97）および 1.65 (95%信頼区間 1.16-2.35)であった。短時間睡眠(6 時間以下)では、全脳卒中の死亡リスクの減少(ハザード比 0.77, 95%信頼区間 0.59-1.01)を示したが、有意差は境界域(P=0.06)であった。全脳卒中と脳梗塞の死亡リスクの量反応関係は、有意であった（それぞれ P<0.0001 および P=0.0002)。脳出血において、7 時間睡眠に比較し、6 時間以下の睡眠では死亡リスクが有意に減少(ハザード比 0.64 (95%信頼区間 0.42-0.98)し、特に男性において、顕著なリスク低下（ハザード比 0.31 (95%CI 0.16-0.64)）を認めた。
【結論】全脳卒中および脳梗塞において、長時間睡眠は死亡リスク増加との関係を示した。脳出血において、男性の短時間睡眠は死亡リスク低下との関係が示唆された。

キーワード：睡眠時間、心血管、脳卒中、脳梗塞、コホート研究
